# Epidemiology of criniviruses: an emerging problem in world agriculture

**DOI:** 10.3389/fmicb.2013.00119

**Published:** 2013-05-16

**Authors:** Ioannis E. Tzanetakis, Robert R. Martin, William M. Wintermantel

**Affiliations:** ^1^Department of Plant Pathology, Division of Agriculture, University of ArkansasFayetteville, AR, USA; ^2^Horticultural Crops Research Laboratory, United States Department of Agriculture-Agricultural Research ServiceCorvallis, OR, USA; ^3^Crop Improvement and Protection Research Unit, United States Department of Agriculture-Agricultural Research ServiceSalinas, CA, USA

**Keywords:** *Crinivirus*, Closteroviridae, whitefly, transmission, detection, control

## Abstract

The genus *Crinivirus* includes the whitefly-transmitted members of the family Closteroviridae. Whitefly-transmitted viruses have emerged as a major problem for world agriculture and are responsible for diseases that lead to losses measured in the billions of dollars annually. Criniviruses emerged as a major agricultural threat at the end of the twentieth century with the establishment and naturalization of their whitefly vectors, members of the genera *Trialeurodes* and *Bemisia*, in temperate climates around the globe. Several criniviruses cause significant diseases in single infections whereas others remain asymptomatic and only cause disease when found in mixed infections with other viruses. Characterization of the majority of criniviruses has been done in the last 20 years and this article provides a detailed review on the epidemiology of this important group of viruses.

## INTRODUCTION

The genus *Crinivirus* is one of the three genera in the family Closteroviridae and includes viruses with segmented genomes, transmitted by whiteflies (Martelli et al., 2011). Details on the molecular biology of the criniviruses are presented in the Kiss et al.(2013) article and for the most part will not be duplicated in this communication. Instead this article will focus on virus epidemiology.

Criniviruses are emerging worldwide, with the first member of the genus, *Beet pseudo-yellows virus* (BPYV) identified in the 1960s (Duffus, 1965). Since then there has been a steady increase in the number of new species with most identified over the past 20 years (Winter et al., 1992; Celix et al., 1996; Duffus et al., 1996a,b; Liu et al., 1997; Wisler et al., 1998a; Salazar et al., 2000; Wisler and Duffus, 2001; Martin et al., 2004; Martín et al., 2008; Tzanetakis et al., 2004; Okuda et al., 2010).

Crinivirus genomic RNAs are encapsidated into long flexuous rods averaging between 650 and 1000 nm in length (Liu et al., 2000; Kreuze et al., 2002), and have large bipartite or tripartite genomes of positive-sense single-stranded RNA totaling approximately 15.3–17.7 kb. Genome organization is similar across the genus, but there are also apparent differences among species. RNA1 encodes proteins that are associated predominantly with replication, whereas RNA2 [or RNAs 2 and 3 for *Potato yellow* vein virus (PYVV)] encodes up to 10 proteins with a range of functions including but not limited to virus encapsidation, cell-to-cell movement, and vector transmission. Most genomic RNAs have common or highly conserved nucleotides at the 5^′^ end ranging from 4 to 11 nucleotides in length. The 3^′^ untranslated regions for each virus other than *Lettuce infectious yellows virus* (LIYV) share a region of approximately 150 nucleotides with a high degree of genetic conservation between the genomic RNAs.

**FIGURE 1:**
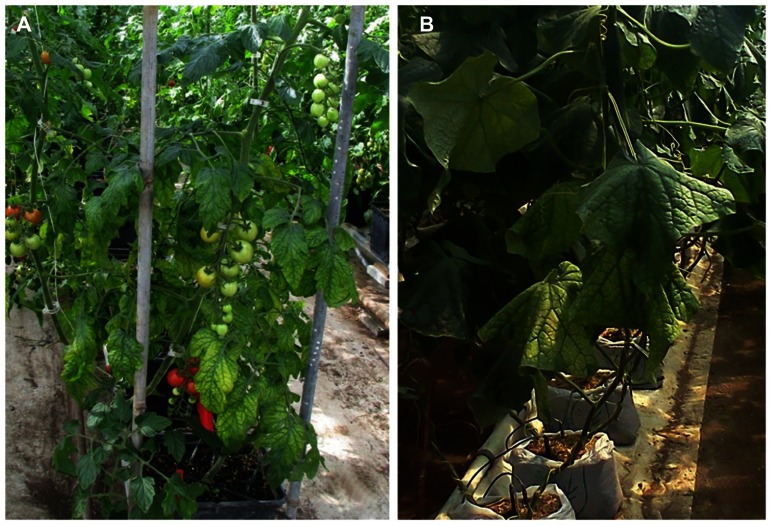
**(A)** Symptoms of *Tomato infectious chlorosis virus* infection, showing interveinal yellowing on middle to lower portions of a tomato plant, while newer growth remains asymptomatic; **(B)** symptoms of mottling and interveinal chlorosis resulting from *Beet pseudo-yellows virus* infection of cucumber. Symptoms are prominent near the crown, less apparent near ends of vines.

Crinivirus transmission is species-specific and performed exclusively by whiteflies in the genera *Trialeurodes* and *Bemisia* in a semi-persistent manner; the reason they are identified with increasing frequency in tropical and subtropical climates where whitefly populations are present. They often cause symptoms that are readily mistaken for physiological or nutritional disorders or pesticide phytotoxicity. Typically, infection is associated with a loss of photosynthetic capability, often characterized by interveinal yellowing of leaves, leaf brittleness, reduced plant vigor, yield reductions, and early senescence, depending on the host plant affected. Some plants may exhibit an interveinal reddening rather than yellowing. Others may exhibit chlorotic mottle on some leaves, usually progressing into interveinal discoloration. Symptoms generally first appear 3–4 weeks after infection, and are most apparent on the older areas of the plant, whereas new growth appears normal. For example, a tomato plant infected with a crinivirus may show extensive interveinal yellowing on leaves near the base, developing interveinal chlorosis on leaves in the middle of the plant, but no symptoms near the apex (**Figure 1**). Similarly, an infected cucumber plant may appear healthy near the growing point of the vines, but exhibit progressively more severe interveinal yellowing toward the crown (**Figure 1**). In both cases it is not uncommon for brittle, symptomatic leaves to snap when bent.

An interesting characteristic of many of the criniviruses studied to date is their ability to interact with other viruses in plants and alter symptoms. Studies have shown host-specific competition between crinivirus species that influence accumulation of other viruses present in the plant and consequently symptom severity (Karyeija et al., 2000; Susaimuthu et al., 2008; Wintermantel et al., 2008). Other viruses interact with distantly related or unrelated co-infecting viruses, resulting in increased disease severity whereas single crinivirus infections may remain asymptomatic (Karyeija et al., 2000; Tzanetakis et al., 2004, 2006b).

Management of criniviruses is predominantly through management of their whitefly vectors. Criniviruses routinely emerge in areas with regularly occurring or persistent whitefly populations, or as vector populations migrate or are moved to new regions. An effective vector control regimen can slow spread or reduce severity of infections; however, such methods will not prevent infection as most criniviruses can be transmitted within the relatively short acquisition and transmission periods of a few hours (Wisler and Duffus, 2001). Sources of host plant resistance have been identified to some criniviruses (McCreight, 1987, 2000; Lopez-Sesé and Gomez-Guillamon, 2000; Aguilar et al., 2006; Eid et al., 2006; Garcia-Cano et al., 2010; McCreight and Wintermantel, 2011) and efforts to identify additional sources are in progress. This may offer potential for effective control and reduced pesticide application as resistance is incorporated into commercial cultivars. Recent studies have also shown that deterrence may effectively reduce whitefly and subsequently virus pressure within fields. For example, acylsucrose expressed through type IV glandular trichomes on tomato have been shown to interfere with the ability of whiteflies to settle and feed steadily, and this can significantly reduce primary and secondary spread of the *Begomovirus*, *Tomato yellow leaf curl virus* (Rodriguez-Lopez et al., 2011, 2012). Although no conclusive studies have been completed with criniviruses, preliminary studies on tomatoes expressing acyl sugars demonstrated delayed *Tomato infectious chlorosis virus* (TICV) symptom development in the field by as much as a month compared with controls (Mutschler and Wintermantel, 2006).

**FIGURE 2:**
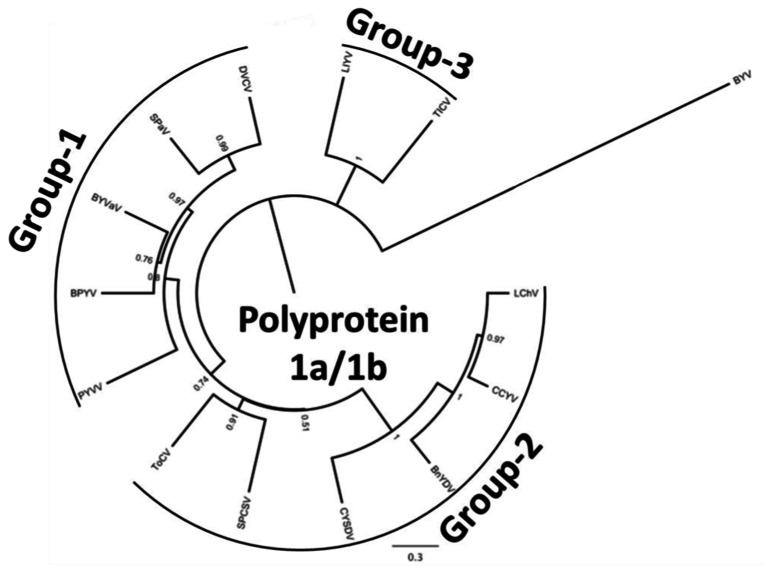
**Phylogenetic analysis of the genus *Crinivirus* based on the 1a/1b fusion polyprotein sequences**. All protein sequences have been obtained from the GenBank genomic sequences of the respective virus. BnYDV, *Bean yellow disorder virus*; BYVaV, *Blackberry yellow vein associated virus*; BPYV, *Beet pseudo-yellows virus*; CCYV, *Cucurbit chlorotic yellows virus*; CYSDV, *Cucurbit yellow stunting disorder virus*; DVCV, *Diodia vein chlorosis virus*; LChV, *Lettuce infectious chlorosis virus*; LIYV, *Lettuce infectious yellows virus*; PYVV, *Potato yellow vein virus*; SPaV, *Strawberry pallidosis associated virus*; SPCSV, *Sweet potato chlorotic stunt virus*; ToCV, *Tomato chlorosis virus*; TICV, *Tomato infectious chlorosis virus*. *Beet yellows virus* (BYV) is used as the outgroup. The bar represents 0.3 amino acid changes/site.

In this communication we provide information on the recent advances in crinivirus epidemiology and associated diseases. Viruses will be presented according to their phylogenetic grouping (Wintermantel et al., 2009b; **Figure 2**) as members of each group tend to have similar vectors and host ranges (**Table 1**).

**Table 1:** *Crinivirus* species and their known vectors.

Virus	Whitefly vector					
	BtA	BtB	BtQ	Baf	Tvp	Tab
*Abutilon yellows virus* (AYV)						X
*Beet pseudo-yellows virus* (BPYV)					X
*Bean yellow disorder virus* (BnYDV)			X		
*Blackberry yellow vein associated virus* (BYVaV)					X	X
*Cucurbit chlorotic yellows virus* (CCYV)		X	X			
*Cucurbit yellow stunting disorder virus* (CYSDV)	X	X	X			
*Diodia vein chlorosis virus *(DVCV)					X	X
*Lettuce chlorosis virus* (LCV)	X	X				
*Lettuce infectious yellows virus* (LIYV)	X					
*Potato yellow vein virus* (PYVV)					X	
*Strawberry pallidosis associated virus* (SPaV)					X	
*Sweet potato chlorotic stunt virus* (SPCSV)		X		X		
*Tomato infectious chlorosis virus* (TICV)					X	
*Tomato chlorosis virus* (ToCV)	X	X	X		X	X

*BtA, Bemisia tabaci biotype A; BtB, B. tabaci biotype B; BtQ, B. tabaci biotype Q; Baf, Bemisia afer; Tab, T. abutilonea; Tvp, Trialeurodes vaporariorum*.

## GROUP-1

### *ABUTILON YELLOWS VIRUS* 

*Abutilon yellows virus* (AYV) is a partially characterized crinivirus originally identified from the common weed velvetleaf (*Abutilon theophrasti* Medic.) collected from Illinois in 1977 (Liu et al., 1997). AYV has flexuous filamentous particles of 12 nm in diameter, approximately 850–900 nm in length (Liu et al., 1997, 2000) but the genome remains uncharacterized; with the exception of the coat protein and a region of the replication-associated polyprotein genes (Liu, unpublished data).

*Abutilon yellows virus* was the first crinivirus known to be transmitted exclusively by *T. abutilonea* Haldeman (banded-wing whitefly) but there is limited information on its host range and its geographic distribution. No crop plants have been identified as hosts; however, the virus can infect members of the Malvaceae, and the experimental solanaceous species, *Nicotiana clevelandii* A. Grey (Liu et al., 1997). AYV symptoms of foliar vein yellowing appear 2–3 weeks after inoculation on the malvaceous weed *Anoda abutiloides* A. Gray (Wisler and Duffus, 2001), symptoms that are highly unusual for criniviruses.

Like other members of the genus, AYV is not mechanically transmissible. To date, the only known vector of AYV remains *T. abutilonea*, and the virus can be retained by the whitefly for up to 3 days (Wisler and Duffus, 2001). Transmission efficiency varied from 4% for individual whiteflies to 81% for 50 whiteflies with acquisition access periods (AAP) of 24 h and inoculation access periods (IAP) of 48 h; whereas efficiency of virus acquisition varied from 19% for single whiteflies to 77% when 50 of the insects were used (Wisler and Duffus, 2001).

### *BEET PSEUDO-YELLOWS VIRUS* 

*Beet pseudo-yellows virus* was first described in 1965 (Duffus, 1965) from sugar beet grown in greenhouses for the sugar beet indexing programs in California and subsequently found to be worldwide in distribution wherever the vector, *T. vaporariorum* Westwood (greenhouse whitefly) is found (Wisler et al., 1998a). The range of *T. vaporariorum* has increased dramatically in recent years with the movement of plant material as has BPYV. Both virus and vector have become serious problems for greenhouse production of vegetables, fruits, and ornamentals worldwide. BPYV is transmitted very efficiently by its vector (Wisler et al., 1998a; Tzanetakis et al., 2006b), a property uncommon among criniviruses (Wintermantel, 2004). Additionally, once introduced into areas where *T. vaporariorum* does well outside the protected environment of greenhouses, the vector has often become naturalized and BPYV often becomes problematic in field-grown crops, as was the case in the western United States (Wintermantel, 2004). Another unique feature of BPYV is its broad host range infecting plants in at least 12 plant families including many vegetable, ornamental, and berry crops. Typical symptoms include interveinal chlorosis as leaves mature (**Figure 1**), reduced growth and fruit size, and early senescence in cucurbits (Wisler et al., 1998a). BPYV was first reported in a rosaceous host, strawberry in 2002 and is one of the criniviruses that can induce strawberry pallidosis disease in *Fragaria virginiana* Duchesne clones UC-10 and UC-11 (Tzanetakis et al., 2003). In California, where the vector has become naturalized, BPYV is now quite common in strawberry (Martin and Tzanetakis, 2013). It was also reported from blackberry in the southeastern United States in plants that exhibited symptoms of blackberry yellow vein disease (BYVD; Tzanetakis and Martin, 2004b). At present, BPYV is rare in blackberry (Tzanetakis, unpublished). If the vector becomes naturalized in the southeastern United States, BPYV will likely become a greater problem in blackberry given that many weed hosts are present in and around blackberry fields in that region (Martin et al., 2013).

Two isolates of BPYV have been fully sequenced, the first from Japan (Hartono et al., 2003), originally named Cucumber yellows virus, that will be referred to as the cucumber isolate here, and a strawberry isolate from the United States (Tzanetakis and Martin, 2004a). The genome size ranges from 15.5 to 15.9 kb with features found in other members of genus; with two or three open reading frames (ORFs) in RNAs 1 and 7 or eight in RNA2. The differences between isolates is noteworthy; the first being a 147 nucleotide insertion after the methyltransferase domain in the replication-associated polyprotein of the strawberry isolate (Tzanetakis and Martin, 2004a). The nucleotide sequence identity of the two isolates before the insertion is 86%, whereas after the insertion the identity is elevated to 94% indicating a possible recombination event. Additionally, the cucumber isolate lacks an ORF at the 3^′^ end of RNA1 that is present in the strawberry isolate. There are also significant differences between the two BPYV isolates on RNA2. RNA2 of the cucumber isolate contains seven ORFs whereas the strawberry isolate has eight. The extra ORF in the strawberry isolate codes for a putative 6 kDa protein with counterparts in several other criniviruses. Based on criteria used to differentiate species in the genus *Crinivirus*, these two isolates of BPYV are clearly distinct strains of the same virus based on amino acid sequence identities of key gene products differing by less than 25% [RNA-dependent RNA polymerase, 98% identical; coat proteins, 99% identical; heat shock protein 70 homolog (HSP70h), 99% identical at the amino acid level; Martelli et al., 2011]. Still, the strawberry strain appears to be the dominant variant in the Americas and as noted affects a wide range of crop and weed hosts (Ramírez-Fonseca et al., 2008; Tzanetakis et al., unpublished).

Because BPYV symptoms are often confused with physiological and nutritional disorders it is likely that the impact and significance of the virus in vegetables and other crops has been underestimated. Additionally, since in most cases symptoms caused by BPYV are those of general plant stress it is important to do virus testing before taking corrective action. Given the great variability among strains, it may be more appropriate to use degenerate primers for virus detection (Wintermantel and Hladky, 2010) that will minimize the possibility of false negatives in testing. To date no sources of resistance have been identified against BPYV.

### *BLACKBERRY YELLOW VEIN ASSOCIATED VIRUS* 

**FIGURE 3:**
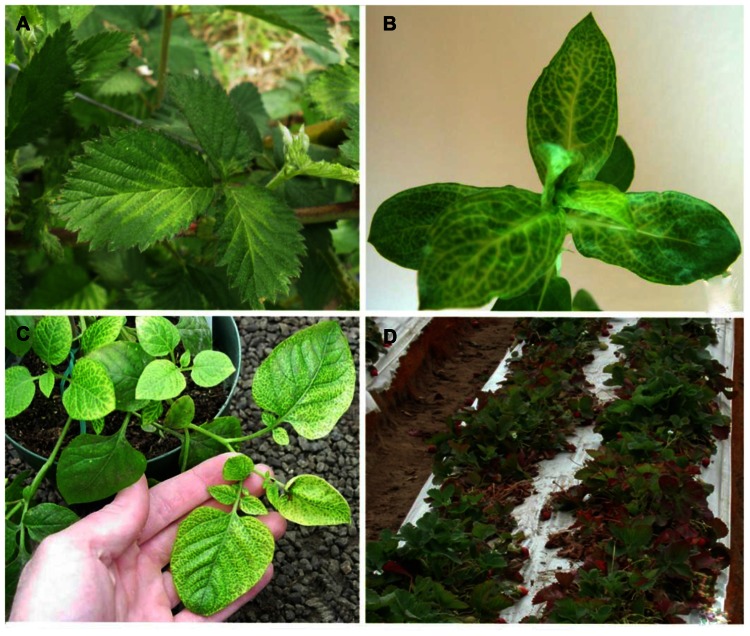
**(A)** Blackberry infected with *Blackberry yellow vein associated virus* and *Blackberry chlorotic ringspot virus* showing symptoms of yellow vein disease; **(B)** *Diodia virginiana* infected with *Diodia vein chlorosis virus* showing vein netting symptoms; **(C)** potato infected with *Potato yellow vein virus* showing yellow vein disease symptoms; **(D)** strawberry decline symptoms of leaf reddening and dieback associated with *Beet pseudo-yellows virus* and *Strawberry pallidosis associated virus* co-infection with other viruses.

Blackberry yellow vein disease was first observed in the North and South Carolina in 2000 and has since become the most important disease affecting blackberry production in the southeastern United States (Martin et al., 2013). Symptoms of BYVD only occur when blackberry plants are infected with more than one virus. Symptoms include vein yellowing, oak-leaf or irregular patterns of chlorosis, ringspots, and line patterns (**Figure 3**; Susaimuthu et al., 2007, 2008). Floricanes can also be severely affected leading to misshapen fruit and cane dieback. In the past, this disease was thought to be caused by *Tobacco ringspot virus* (TRSV) as this was the only virus that was mechanically transmissible from plants with such symptoms. This was questioned when blackberry plants were infected with TRSV using nematodes and infected plants did not develop symptoms over a 3-year period. The first virus characterized from blackberry plants that exhibited BYVD symptoms from South Carolina was a crinivirus, and named *Blackberry yellow vein associated virus* (BYVaV; Martin et al., 2004).

*Blackberry yellow vein associated virus* is a typical crinivirus with a bipartite genome. RNA1 is 7.8 kb in length and encodes only the replication-associated polyprotein whereas RNA2 is about 7.9 kb and contains the eight ORFs typical of other criniviruses. BYVaV RNA2 contains an additional ORF at the 5^′^ end that encodes for a second transmembrane protein, that is absent from RNA2 of other criniviruses (Tzanetakis et al., 2006a).

Once detection primers were developed it was observed that BYVaV could be detected in both symptomless and symptomatic plants of several blackberry cultivars, suggesting a complex etiology for BYVD. Since that time multiple viruses have been characterized from blackberry with BYVD symptoms and in all cases symptomatic plants had mixed virus infections (Martin et al., 2013). BYVaV is the most common virus found in plants that exhibit BYVD symptoms. BYVaV does not cause symptoms on the standard woody indicators used for graft indexing in *Rubus* certification programs, which explains its presence in nursery stocks prior to the development and application of PCR-based detection assays (Susaimuthu et al., 2007). Studies with several isolates of BYVaV from cultivated and wild blackberry from diverse geographic areas showed diversity at the nucleotide level as high as 12% and suggested that recombination between isolates is likely a factor in the evolution of the virus (Poudel et al., 2012). In addition, the study on virus diversity has resulted in the development of a set of detection primers based on conserved sequences from all isolates studied, whereas previous detection primers did not detect all of these isolates (Poudel et al., 2013).

*Blackberry yellow vein associated virus* can be transmitted efficiently from blackberry to blackberry with efficiencies of approximately 50% for *T. abutilonea* and 25% for *T. vaporariorum* when 50 whiteflies were used for inoculation following 18–24 h AAP and 48 h IAP (Poudel et al., 2013). BYVaV was not detected in any of 25 plant species that were common in or near blackberry fields with a high incidence of BYVaV infection. Even though BYVaV could be graft transmitted to rose, it was not detected in 40 samples of rose tested in native settings with high BYVaV pressure (Poudel et al., 2013) suggesting that wild rose likely is not an important component of the epidemiology of BYVaV. The virus has been detected throughout the southeastern United States, in California and Oklahoma and as far north as Illinois and West Virginia, but with surprisingly low incidence in Georgia and Florida. Overall, 145 of 234 samples of cultivated and native blackberries that exhibited BYVD symptoms tested were positive for BYVaV (Poudel et al., 2013). Given the complexity of BYVD there have not been efforts to introduce resistance for to BYVaV.

### *DIODIA VEIN CHLOROSIS VIRUS* 

Virginia buttonweed (*Diodia virginiana* L.) is a member of the Rubiaceae (coffee family). Its natural habitat is in wetlands of the Americas, extending between the 45th parallels of both continents. It propagates in a prolific manner through stolons and seed, making it one of the most noxious weeds of turfgrass. Several Virginia buttonweed populations in the southern United States show distinct vein chlorosis or vein netting symptoms, typical of virus infection (**Figure 3**). Larsen et al. (1991) studied the disease and discovered virus aggregates in infected material similar to those found in closterovirus-infected plants. The putative virus produced double-stranded RNA was similar in size to that of LIYV and *T. abutilonea* was experimentally verified as a vector. All these properties indicated that *Diodia vein chlorosis virus* (DVCV) is a member of the genus *Crinivirus* but no molecular information was available until recently when an isolate from a clone of a plant used in the Larsen et al.’s (1991) study was sequenced (Tzanetakis et al., 2011). DVCV genome is composed of 16.2 kb with RNA1 coding for the replication-associated polyprotein and RNA2 having the normal array of eight genes found in most members of the genus. Phylogenetic analysis clearly placed DVCV in group-1 of the genus. Given that all members of the group have been proven transmissible with *T. vaporariorum*, this was evaluated for DVCV. Indeed, both *T. abutilonea* and *T. vaporariorum* transmit the virus with efficiencies of over 36 and 12%, respectively when plants were inoculated with 50 whiteflies after 48-h AAP and IAP (Tzanetakis et al., 2011). The phylogenetic placement of DVCV, its vectors and the co-habitat of *D. virginiana* and berry crops resulted in a decision to conduct a series of experiments to determine the ability of the virus to infect strawberry and blackberry. Those experiments failed to identify additional hosts for DVCV other than *D. virginiana*. Given that the only known host for DVCV is a weed, control measures are not employed for this virus other than the elimination of Virginia buttonweed through the use of herbicides.

### *POTATO YELLOW VEIN VIRUS* 

Potato, a plant native to South America, is a host of several viruses. Many are asymptomatic in single infections, and as many cause devastating diseases that lead to major losses (Salazar, 2006). Plants affected by potato yellow vein disease can suffer losses reaching as much as 50%. Typical symptoms include vein yellowing that gives leaves the appearance of a yellow net (**Figure 3**). The disease was first identified in 1943 and has since been reported in Venezuela, Columbia, Ecuador, and Peru (Diazmore, 1963; Salazar, 2006). It was not until the turn of the century that the putative causal agent was identified and characterized (Salazar et al., 2000). The agent was transmitted by *T. vaporariorum* and named PYVV. Virus purifications and cloning of the HSP70h gene of the virus indicated that is a member of the genus *Crinivirus* (Salazar et al., 2000). PYVV is a unique crinivirus as the only member of the genus with a tripartite genome. RNA1 is organized similarly to other criniviruses, encoding the replication-associated proteins and small peptide with a transmembrane domain. RNA2 encodes five proteins that are found in the 5^′^ terminus of the crinivirus orthologous molecule whereas RNA3 has three ORFs commonly found at the 3^′^ terminus of RNA2 in other crinivirus species, indicating that PYVV is probably a product of an ancestral virus segmentation in which the ancestral RNA2 segregated into PYVV RNAs 2 and 3 (Livieratos et al., 2004) although, in phylogenetic terms, it appears ancestral to the bipartite members of group-1 (**Figure 2**). The host range of the virus is rather restricted, and includes species in the genera *Solanum*, *Polygonum*, *Rumex*, *Tagetes*, *Catharanthus*, and *Malva* able to sustain virus replication whereas many common crinivirus indicators including *Nicotiana*, *Datura*, and *Physalis* species are resistant to infection (Salazar et al., 2000; Guzman and Rodriguez, 2010). The limited host range of the virus is reinforced by the fact that studied isolates present rather limited diversity (Offei et al., 2004; Guzmán et al., 2006; Rodriguez-Burgos et al., 2010). PYVV is closely associated with yellow vein disease symptoms but Koch’s postulates have not been fulfilled as the virus can remain asymptomatic in potato. The importance of the disease, the ease of transmission, as recorded with the transmission of the virus in greenhouses in the UK, in combination with the asexual propagation and the cosmopolitan growth of the potato industry has made the development of advanced detection methods obligatory for the industry, and there are reports of sensitive detection protocols available (López et al., 2006). Virus control is based on insecticide use and strict quarantine directives that would not allow virus spread outside the countries where it is already present.

### *STRAWBERRY PALLIDOSIS ASSOCIATED VIRUS* 

Strawberries (family Rosaceae) are known to be natural hosts for about 30 viruses (Martin and Tzanetakis, 2006; Tzanetakis, 2010 ), several of which occur wherever the crop is grown and can cause significant losses (Spiegel and Martin, 1998). Pallidosis disease initially was identified in the United States during the 1950s (Frazier and Stubbs, 1969). Symptoms on indicator plants of *F. virginiana* clones “UC-10” or “UC-11,” can include leaf distortion, chlorosis, and some dwarfing, though under less than optimal conditions for symptom development it is easy to overlook symptoms. Two viruses have been consistently associated with the disease, BPYV and *Strawberry pallidosis associated virus* (SPaV). Sequencing of the genome of SPaV confirmed it as a crinivirus (Tzanetakis et al., 2005). It contains two RNAs, both approximately 8 kb, with typical crinivirus genome organization. SPaV is most closely related to BPYV and AYV based on phylogenetic analysis (Tzanetakis et al., 2005).

There have been reports of severe strains of the pallidosis agents that are lethal on indicators. Graft transmission of multiple isolates from the eastern and western United States caused only mild symptoms and it is most likely that these “severe strains” actually represented mixed virus infections involving not only a crinivirus, but likely another partner virus (Hokanson et al., 2000; Tzanetakis et al., 2004).

*Strawberry pallidosis associated virus* is transmitted by *T. vaporariorum*, although somewhat inefficiently compared to BPYV (Tzanetakis et al., 2006b). Surprisingly, SPaV was more common in strawberry than BPYV in field settings. Both viruses were found in the majority of plants that exhibited decline symptoms due to mixed virus infections in California in the 2002–2003 periods (**Figure 3**). The decline epidemic was estimated to cause losses of about 50 million dollars for the two seasons (Martin and Tzanetakis, 2013). Plants were infected with at least one of the two criniviruses (BPYV or SPaV) and one of the common aphid-transmitted strawberry viruses (*Strawberry crinkle virus*, *Strawberry vein banding virus*, *Strawberry mottle virus*, or *Strawberry mild yellow edge virus*); incidence of SPaV was as high as 90% compared to 40% for BPYV. In plants from the Mid-Atlantic states that indexed positive for pallidosis disease based on symptoms, 37 of 38 plants were positive for SPaV and only about 25% were positive for BPYV (Tzanetakis et al., 2006b). Either virus can cause pallidosis symptoms in indicator plants. In other comparisons, SPaV was always more common in strawberry plants in side-by-side field comparisons than BPYV. This suggests that in nature there are other factors that contribute to virus transmission efficiency than what is typically measured in greenhouse or growth chamber studies. It is possible that the colony of whiteflies used in the greenhouse studies is better adapted to transmission of BPYV than SPaV or there are other, yet to be identified, vectors that are more efficient for transmission of SPaV. SPaV had a very limited host range in greenhouse studies, where it did not infect *Urtica urens* L., but was found in an *Urtica* species in the field in an area with high *T. vaporariorum* populations, though this could have been a different *Urtica* species (Tzanetakis et al., 2006b). The virus has been reported in strawberry production areas throughout the Americas, Australia, and Egypt (Wintermantel et al., 2006; Ragab et al., 2009; Constable et al., 2010; Martin and Tzanetakis, 2013). Both BPYV and SPaV are asymptomatic in single or mixed infections in “Hood” and “Noreaster” strawberry (Tzanetakis, 2004). Given the annual plasticulture that has been adapted in most production areas in the world it is imperative that plants do not become infected within the nursery system. Infections with the strawberry criniviruses may be asymptomatic but when plants accumulate additional viruses in the field, they can decline rapidly. The titer of SPaV declines in summertime and for this reason testing for this virus is recommended in spring or late fall using younger but fully expanded leaves (Tzanetakis et al., 2004). As in the case of BYVaV, the symptomless single infections and the complexity of disease-causing virus complexes have discouraged work toward identification of accessions which preclude virus replication.

## GROUP-2

### *BEAN YELLOW DISORDER VIRUS* 

Legumes (family Fabaceae) are infected by numerous viruses, several of which cause significant losses with many regularly identified in new locations around the world (de Oliveira et al., 2011; Zhou et al., 2011). This was also the case of a disease observed in common bean (*Phaseolus vulgaris* L.) in Spain in 2003. Symptoms were similar to nutritional disorders with yellowing of the leaf blade, whereas pods appeared malformed. Leaves were brittle and whitefly transmission with *B. tabaci* Gennadius yielded reproducible symptoms. These observations pointed to a crinivirus infection. Confirmation came with the cloning of the HSP70h gene of the virus, which was named *Bean yellow disorder virus* (BnYDV; Segundo et al., 2004). An extended study in greenhouses in Spain, the only country the virus is known to exist, showed BnYDV incidence of about 6%, indicating that the virus was an emerging problem for bean growers (Segundo et al., 2008). BnYDV genome is 17.5 kb; encoding four proteins in RNA1 and nine in RNA2 (Martín et al., 2008). Phylogenetic analysis indicated the close relationship of BnYDV with vegetable-infecting criniviruses that are efficiently transmitted by *B. tabaci* (Martín et al., 2008). Transmission experiments revealed efficiencies that exceeded 35% using single whiteflies with 24 h AAP and IAP, respectively. A much more surprising result was the retention ability of *B. tabaci* which reached 2 weeks when most other criniviruses are retained for less than a week (Martín et al., 2011). More than 30 species belonging to the families Asteraceae, Boraginaceae, Cucurbitaceae, Fabaceae, Geraniaceae, Lamiaceae, Malvaceae, Scrophulariaceae, Solanaceae, Thymelaeaceae, and Verbenaceae were evaluated as hosts but only four legume species (*P. vulgaris* L., *Pisum sativum* L., *Lens culinaris* Medik., and *Vicia faba* L.) were able to sustain virus replication. Given the high incidence of the virus in greenhouses, control measures have primarily focused in these production systems. Beans grown in screenhouses had 14 times fewer whiteflies per plant. The incidence of the virus under screenhouse protection never exceeded 12.5% unlike that in conventional greenhouses which reached over 80% (Janssen et al., 2011). Given the incidence of the virus in the confined environment of a greenhouse, the physical barrier of fine mesh screenhouses appears to be the most efficient approach to minimize vector presence and associated virus transmission.

### *CUCURBIT CHLOROTIC YELLOWS VIRUS* 

Cucurbits are grown throughout the world and are exposed to a wide array of production environments and pests. These crops are known to be infected by more than 60 viruses (Lecoq and Desbiez, 2012), and several are discovered each year (Brown et al., 2011; Lecoq et al., 2011; Dong et al., 2012). Melon plants with severe yellowing symptoms in Kumamoto, Japan tested negative for known cucurbit viruses. Further research revealed that the disease agent was transmissible with *B. tabaci* biotypes B and Q whereas limited sequence data revealed that the agent shared similarities with criniviruses (Gyoutoku et al., 2009). The virus, now known as *Cucurbit chlorotic yellows virus* (CCYV), has a typical bipartite crinivirus genome, encoding four proteins in RNA1 and eight in RNA2 (Okuda et al., 2010). Phylogenetic analysis revealed the placement of CCYV into group-2. Okuda et al. (2010) studied the ability of the virus to replicate and move systemically in 19 additional hosts belonging to the families Asteraceae, Chenopodiaceae, Convolvulaceae, Cucurbitaceae, Fabaceae, and Solanaceae. The majority were shown to accommodate systemic movement, expanding the known CCYV host range. Since its first report in 2004, CCYV has spread to Taiwan, China, North Africa, and the Middle East, always found in association with severe disease outbreaks in cucurbits (Huang et al., 2010; Gu et al., 2011; Hamed et al., 2011; Abrahamian et al., 2012). Virus infection can significantly reduce crop characteristics in melon and watermelon, with significant brix reduction and yield losses that can reach a third of the crop when virus incidence is higher than 75% (Peng and Huang, 2011). Gyoutoku et al. (2009) have developed an efficient RT-PCR test for the virus but the widespread presence of the virus led to the need for high-throughput detection protocols. For this reason, Kubota et al. (2011) developed antibodies against the recombinant coat protein able to detect the virus using immunoelectron microscopy, tissue blot and ELISA. The importance of the virus and the significant yield losses have led to efforts toward identification of resistance in melon with five accessions from the Indian subcontinent exhibiting promising results (Okuda et al., 2013). Until resistance is incorporated into commercial cultivars, control will require insecticide treatment of whitefly-infested areas.

### *CUCURBIT YELLOW STUNTING DISORDER VIRUS* 

*Cucurbit yellow stunting disorder virus* (CYSDV) was initially discovered in the United Arab Emirates in 1982 (Hassan and Duffus, 1991). Virus particles range from 825 to 900 nm in length (Celix et al., 1996), and the two RNAs are 9.1 and 8 kb, with genome organization similar to other criniviruses.

**FIGURE 4:**
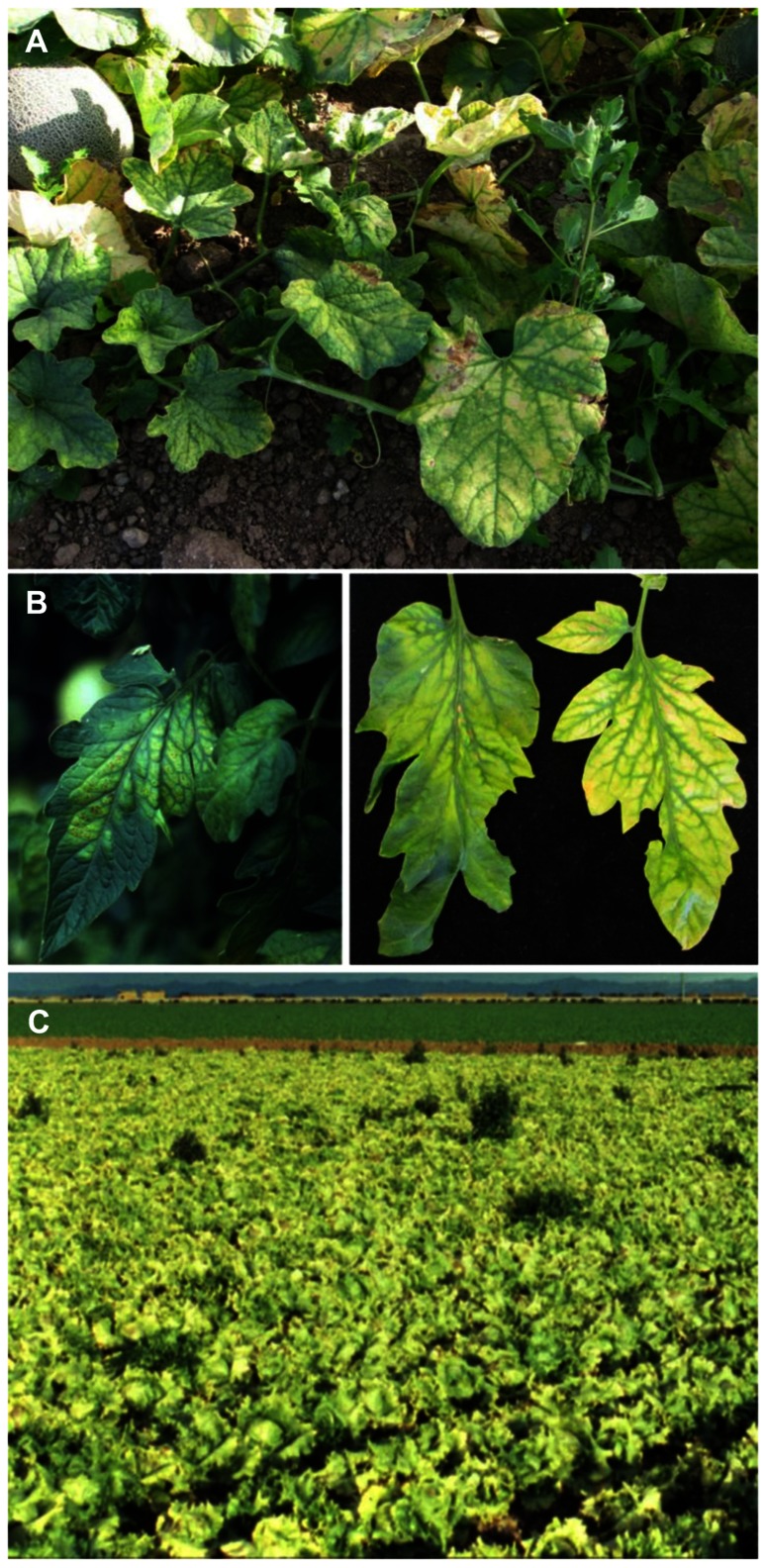
**(A)** Severe interveinal chlorosis in melon caused by *Cucurbit yellow stunting disorder virus*; **(B)** typical symptoms of *Tomato chlorosis virus* (left) and *Tomato infectious chlorosis virus* (right) on tomato leaflets, illustrating the range of similar symptoms produced by both viruses; **(C)** lettuce field exhibiting classic yellowing symptom due to *Lettuce infectious yellows virus*.

*Cucurbit yellow stunting disorder virus* has been very successful in spreading from the Middle East to many cucurbit production regions throughout the world. Affected production regions include, in addition to the Middle East, the Mediterranean Basin including Lebanon, Israel, North Africa, and Southern Europe as well as the Canary Islands (Celix et al., 1996; Wisler et al., 1998a; Abou-Jawdah et al., 2000; Desbiez et al., 2000; Kao et al., 2000; Louro et al., 2000). The virus has recently become a significant production threat throughout cucurbit production regions in the southern United States, Mexico, and Central America. CYSDV is latent for up to 3 weeks but when symptoms develop they appear similar to those of other whitefly-transmitted viruses on cucurbits, with mottle symptoms early followed by extensive interveinal chlorosis (**Figure 4**). As with other criniviruses, symptoms are more prominent on older leaves with younger leaves remaining symptomless. CYSDV infections result in reduced plant vigor, and can significantly reduce fruit sugar production, resulting in poor tasting, unmarketable fruit.

The host range of CYSDV was originally believed to be restricted to members of the Cucurbitaceae (Celix et al., 1996); however, more recent studies have demonstrated CYSDV can infect plant species from at least nine families (Wintermantel et al., 2009a). Although cucurbits are the predominant and most significant agricultural hosts of the virus, common bean can be severely affected, resulting in severe stunting and virtual elimination of yield when infected at an early age. Lettuce is another host of the virus (Wisler et al., 1998a), and can be a reservoir for transmission to other crops, but symptoms are mild and agronomically insignificant (Wintermantel et al., 2009a). Numerous common weeds are also hosts of the virus, but in most cases these plants are symptomless and vary in their ability to serve as effective virus reservoirs for transmission to crop hosts (Wintermantel et al., 2009a).

CYSDV is transmitted very efficiently by at least three biotypes of *B. tabaci* A, B, and Q (Wisler et al., 1998a; Berdiales et al., 1999). The A biotype has become rare after its displacement from its native range in the American Southwest by the B biotype. Both B and Q biotypes are prevalent in many significant cucurbit production regions of the world, and are highly efficient in transmission. When vector populations are high it is virtually impossible to prevent infection of cucurbits. When CYSDV emerged in the American Southwest nearly all cucurbit production was affected during the first year due to the presence of excessively high vector populations.

Studies conducted on isolates collected over geographically distinct regions (Rubio et al., 2001) as well as local populations (Marco and Aranda, 2005), demonstrated most isolates are highly conserved genetically. Proteins show significant variation and among them the coat protein region seems to exhibit the most substantial variability, illustrating the divergence of a cluster of isolates from Saudi Arabia from other isolates identified from throughout the world (Rubio et al., 2001). Examination over time of a CYSDV collection from a localized region in Spain demonstrated an exceptionally high level of conservation within the virus population compared with other plant viruses (Marco and Aranda, 2005). It is speculated that genetic bottlenecks may influence the low genetic diversity within local populations. Similarly, genetic bottlenecks may also influence emergence of unique variants as observed for Arabian isolates (Marco and Aranda, 2005).

Management of CYSDV is predominantly through insecticide based vector control, which reduces vector numbers and results in slower rates of symptom development, but does not prevent virus transmission. Increasing efforts are focusing on development of virus resistance, particularly in cucumber and melon (Lopez-Sesé and Gomez-Guillamon, 2000; Marco et al., 2003; Aguilar et al., 2006; Eid et al., 2006; McCreight and Wintermantel, 2011), in which new sources of resistance to the virus have been identified in recent years. Efforts are progressing toward characterization of resistance in both hosts and toward combining resistance sources in melon.

### *LETTUCE CHLOROSIS VIRUS* 

Yellowing symptoms, normally associated with the crinivirus, LIYV, were observed in vegetable fields in the southwestern United States in the 1990s. At that point in time LIYV had virtually been eliminated following displacement of its primary vector, *B. tabaci* biotype A. This fact lead Duffus et al. (1996b) to investigate the possibility that other viruses might be present in the region, and ultimately to the discovery of *Lettuce chlorosis virus* (LChV). The virus is transmitted by *B. tabaci* biotypes A and B with similar efficiencies. Whiteflies can acquire and transmit the virus with AAP/IAP of 1 h each. Transmission was more efficient after 24 h of feeding whereas retention did not exceed 4 days. The host range includes at least 31 species belonging to 13 families, with several noteworthy hosts including spinach, sugar beet, and several weed species commonly found in the southwestern United States (Duffus et al., 1996b; McLain et al., 1998). The two genomic RNAs of the virus are contain in individual particles of 800–850 × 12 nm. The 17-kb genome is arranged similarly to that of other members of group-2, encoding four proteins in RNA1 and 10 in RNA2 (Salem et al., 2009). Insecticide applications can minimize virus incidence, something that is particularly important in early season where LChV can have a significant impact in lettuce yield (McLain et al., 1998). Infected lettuce can exhibit foliar yellowing, but also head deformation if infection occurs early. LChV has not spread to areas outside the southwest United States and is not usually a significant production threat, probably as a result of lettuce-free periods and the inability of the virus to infect other significant crop hosts during the fall season when whitefly populations are elevated.

### *SWEET POTATO CHLOROTIC STUNT VIRUS* 

Sweet potato is one of the most nutritious vegetables, rich in vitamins and microelements and one of the most important staple foods available today in sub-Saharan Africa (Loebenstein and Thottappilly, 2009). Virus-like diseases of sweet potato have been reported for more than 50 years in Africa with several aphid-borne and whitefly-borne agents known to cause significant losses (Schaefers and Terry, 1976). Schaefers and Terry (1976) provided the first evidence that one of the components of the sweet potato virus disease (SPVD), the most important sweet potato disease in sub-Saharan Africa was whitefly-transmitted (Chavi et al., 1997; Gibson et al., 1998; Ateka et al., 2004). About 25 years later the virus, named *Sweet potato chlorotic stunt virus* (SPCSV) was partially characterized at the biochemical level (Winter et al., 1992) and a decade later was fully characterized at the molecular level (Kreuze et al., 2002). SPCSV is the crinivirus with largest genome identified to date with particles of 900–1000 nm in length and two genomic RNAs exceeding 17.6 kb (Winter et al., 1992; Kreuze et al., 2002). RNA1 encodes the replication-associated polyprotein and two or three additional genes depending on the isolate, similar to what is observed for BPYV (Cuellar et al., 2011a). RNA2 has similar architecture to most criniviruses with seven ORFs speculated to be involved in assembly and movement. SPCSV is transmitted by *B. tabaci*, *B. afer* sensu lato, and *T. vaporariorum* (Sim et al., 2000; Gamarra et al., 2010) and has spread to most areas where sweet potato is grown (Yun et al., 2002; Lozano et al., 2004; Abad et al., 2007; Qiao et al., 2011).

While SPCSV appears to exhibit minimal yield effects in single infections as is also the case for some of the other criniviruses presented here, it has a major effect when occurring together with *Sweet potato feathery mottle virus* or other potyviruses, resulting in SPVD. In a seminal paper by Karyeija et al. (2000) it was shown that co-infection of the two viruses leads to a 600-fold titer increase of the *Potyvirus* and subsequent development of SPVD symptoms. It was later shown that similar effects can be observed when the virus exists in mixed infections with viruses of other genera and families, further signifying the importance of the SPCSV in SPVDs (Untiveros et al., 2007; Cuellar et al., 2011b).

There have been several studies on the population structure of SPCSV (Alicai et al., 1999; Fenby et al., 2002; Tairo et al., 2005). There are distinct populations of the virus that show diversity in excess of 25% at the nucleotide level although there is less diversity at the amino acid level. Those studies have identified distinct virus populations, also reinforced by the variability in gene numbers between isolates (Cuellar et al., 2011a), indicating that SPCSV presents a polyphyletic evolutionary pattern.

Given the asymptomatic infection of SPCSV in single infections and its importance in SPVD sensitive cultivars, efficient detection protocols are important for testing propagation stock and minimizing virus movement to areas where the virus is absent. For this reason there are several reports of detection protocols for the virus, both immunological and molecular (Kokkinos and Clark, 2006; Opiyo et al., 2010). There has also been extensive work on identification of resistance for the viruses involved in SPVD using traditional and modern approaches with promising results (Karyeija et al., 1998; Mwanga et al., 2002; Kreuze et al., 2008; Miano et al., 2008). Still, the complexity of the disease and the apparent diversity of the virus make incorporation of viable resistance into commercial cultivars a challenging undertaking.

### *TOMATO CHLOROSIS VIRUS* 

*Tomato chlorosis virus* (ToCV) was originally identified in 1996 from greenhouse-grown tomatoes (*Lycopersicon esculentum* Mill.) from Florida (Wisler et al., 1998b), and exhibits a moderate host range of at least 24 plant species from seven different families (Wintermantel and Wisler, 2006). Symptoms on tomato include interveinal chlorosis, leaf brittleness, and limited necrotic flecking or leaf bronzing, and are nearly identical to those associated with infection by TICV (**Figure 4**), although genetically the two viruses vary significantly. Several methods are now available to differentiate ToCV from TICV, including RT-PCR (Wintermantel and Hladky, 2010; Papayiannis et al., 2011), molecular probes (Garcia-Cano et al., 2010), or virus-specific antiserum (Duffus et al., 1996; Jacquemond et al., 2009; Wintermantel, unpublished).

The 16.8 kb genome of ToCV is typical of criniviruses and is encapsidated as long flexuous virions approximately 800–850 nm in length (Liu et al., 2000). RNA1 encodes four ORFs including proteins associated with virus replication, and suppression of gene silencing (Wintermantel et al., 2005; Cañizares et al., 2008), and RNA2 encodes up to nine ORFs encoding proteins involved in a multitude of functions including virus encapsidation, cell-to-cell movement, membrane association, and whitefly transmission (Stewart et al., 2010; Chen et al., 2011).

The host range of ToCV extends, in addition to tomato, to other solanaceous hosts including pepper (Lozano et al., 2003), potato (Fortes et al., 2012), and tomatillo (Trenado et al., 2007). Several weed species can also harbor ToCV (Font et al., 2004; Wintermantel and Wisler, 2006), and the presence of weed hosts near production areas can provide an alternate host for the virus between cropping seasons, as well as providing an acquisition source for whitefly vectors that can carry the virus back to cultivated hosts.

*Tomato chlorosis virus* is unique among members of the genus as transmission by at least five different whiteflies has been documented (Navas-Castillo et al., 2000; Wintermantel and Wisler, 2006). The virus AAP is short, but transmission occurs more readily when vector whiteflies have IAP of several hours. Transmission efficiency varies among whitefly species, with *T. abutilonea* and *B. tabaci* biotype B, highly efficient vectors, yielding high rates of transmission, whereas *B. tabaci* biotype A and *T. vaporariorum* transmit ToCV with much lower efficiency (Wintermantel and Wisler, 2006). *B. tabaci* biotype Q is also an efficient vector, and has emerged as the predominant vector in southern Europe (Navas-Castillo et al., 2000). Each vector also differs in its ability to retain the virus, with *T. abutilonea* able to transmit for up to 5 days following virus acquisition, whereas *B. tabaci* biotype B loses its ability to transmit ToCV after 3 days. *B. tabaci* biotype A and *T. vaporariorum* lose their transmissibility after only a day (Wintermantel and Wisler, 2006). ToCV has a relatively long latent period in infected host plants, often not inducing symptoms until 3 weeks after infection. If nursery plants are exposed to viruliferous vector populations at an early age, it is possible for ToCV-infected plants to be carried to new areas through movement of transplants prior to symptom development.

Management of ToCV is primarily through the management of vector populations using both chemical and cultural practices. Since criniviruses cannot spread without whitefly vectors, suppression of vector populations can keep crinivirus spread to a minimum. Although insecticides can reduce whitefly populations, such control methods are inefficient for virus control, since whiteflies can transmit viruses before being killed by an insecticide. In addition to vector control, it is important to limit availability of alternate host plants that can serve as virus reservoirs. Testing of nursery stock and ornamental host plants for the presence of these viruses can also reduce movement of ToCV to new areas. Importantly, resistance to ToCV was recently identified in crosses between *Solanum lycopersicum* (tomato) and *S. peruvianum* L., as well as *S. chilense* (Dunal) Reiche (Garcia-Cano et al., 2010). Introgression of this resistance into cultivated tomato should greatly strengthen future management of ToCV.

## GROUP-3

### *LETTUCE INFECTIOUS YELLOWS VIRUS* 

*Lettuce infectious yellows virus* is the most thoroughly studied virus in the genus *Crinivirus*. It was discovered in the southwestern desert agricultural regions of the United States in 1981 (Duffus and Flock, 1982), and was the first crinivirus sequenced (Klaassen et al., 1995). Its 15.3 kb genome partially defined the characteristics of the genus.

*Lettuce infectious yellows virus* has a relatively large host range, infecting at least 45 species of plants in 15 families, and caused significant yield losses for lettuce, melon, and sugar beet. LIYV causes interveinal yellowing symptoms in melon and sugar beet, and a severe yellowing symptom on lettuce that gave the virus its name and resulted in widespread field yellowing (**Figure 4**). Unlike most other criniviruses affecting commercial agriculture, which have effectively been distributed around the world, LIYV remained predominantly confined to southwestern United States and northern Mexico. This is due to its close relationship with the *B. tabaci* biotype A, which shared a common geographical range with the virus (Brown and Nelson, 1986; Duffus et al., 1986). The virus persisted in the region throughout the 1980s, but quickly faded from prevalence with the emergence of the *B. tabaci* biotype B in the early 1990s (Cohen et al., 1992; Brown et al., 1995). As the B biotype became established, the A biotype gradually disappeared from fields, and along with it LIYV. Studies have shown a biological basis for this, with LIYV exhibiting over 100 times greater transmission using the *B. tabaci* biotype A than biotype B (Wisler and Duffus, 2001). LIYV has not been identified in the American Southwest for well over a decade, and although it is possible the virus may still exist in long-term reservoir hosts, the likelihood that it would reemerge is slim, since it is transmitted poorly by current *B. tabaci* biotypes, and the A biotype is no longer present in the field.

### *TOMATO INFECTIOUS CHLOROSIS VIRUS* 

*Tomato infectious chlorosis virus* was discovered in tomato from southern California in 1993 (Duffus et al., 1996a) and has since been identified as a problem for tomato production in many parts of the world including Mexico, Europe, the Middle East, as well as East and Southeast Asia (Wintermantel et al., 2009b). Symptoms on tomato include, similarly to ToCV, interveinal yellowing (**Figure 4**) with leaves becoming thickened and crispy, breaking easily when bent. Yield is affected through decreased fruit size and number (Wisler et al., 1996), as well as decreased plant longevity (Wintermantel, 2004).

*Tomato infectious chlorosis virus* virions consist of long flexuous rods varying from 850 to 900 nm in length (Liu et al., 2000) containing the two RNAs of about 8.3 and 7.9 kb. Similarity between TICV and other criniviruses varies throughout the genome but TICV is related much more closely to LIYV than to any other crinivirus, and together the two form a distinct clade within the genus (Wintermantel et al., 2009b).

The virus is transmitted exclusively by *T. vaporariorum* (Duffus et al., 1996a). TICV can be acquired and transmitted after a 1-h AAP; however, transmission efficiency increases steadily with longer AAPs. A 48-h AAP using 30 whiteflies per plant was most efficient and resulted in 94% transmission. Individual whiteflies given a 24-h AAP on infected source plants transmit TICV at an 8% rate; whereas an 83% transmission rate is found when plants are exposed to 40 viruliferous whiteflies each. Transmission by viruliferous whiteflies also varies over time with transmission using 30 viruliferous whiteflies per plant increasing from 16% transmission with 1 h transmission access periods to 80% when whiteflies are exposed to test plants for 48 h. TICV can persist in whiteflies for up to 4 days, but transmission efficiency drops off dramatically after 24 h (Duffus et al., 1996a).

Although tomato is considered the principal host of TICV, the virus also infects a number of important vegetable and ornamental host plants (Duffus et al., 1996a; Wisler et al., 1996). Lettuce, potato, petunia, artichoke, ranunculus, and China aster can also be infected by TICV. Like other criniviruses, TICV symptoms take up to 3 weeks to develop, and during this period movement of infected plant material by the nursery industry or by commercial vendors can be responsible for distribution of TICV to new regions (Wisler et al., 1998a). The virus can survive during non-crop seasons in a wide range of weed hosts near production areas and move into crops as whitefly populations develop and become active. Similarly, some ornamentals or alternate crops such as lettuce can serve as reservoirs for virus transmission to tomato (Duffus et al., 1996a; Wisler et al., 1998a; Font et al., 2004).

Management of TICV, like other criniviruses, involves both chemical and cultural practices. Since criniviruses cannot spread without whitefly vectors, suppression of vector populations can keep crinivirus spread to a minimum. In addition to vector control, it is important to limit availability of alternate host plants that can serve as virus reservoirs. Although insecticides can reduce whitefly populations, such control methods are inefficient for virus control, since whiteflies can transmit viruses before being killed by an insecticide. Resistance to TICV is not available in cultivated tomato; however, preliminary studies have indicated resistance to whitefly feeding can slow TICV disease progress in cultivated tomato (Mutschler and Wintermantel, 2006).

## DISCUSSION

Closteroviruses cause diseases of great economic importance. *Citrus tristeza virus* has changed the map of citrus production around the world and the Grapevine leafroll associated viruses have had a major impact on vine health and wine quality, both affecting multi-billion dollar industries worldwide. Criniviruses have recently emerged as major pathogens in world agriculture, primarily because of the movement and establishment of their whitefly vectors in temperate regions around the world.

There are clear cases in which criniviruses are the causal agents of devastating diseases such as CYSDV and BPYV in cucurbits or TICV and ToCV in tomato. In addition, there are many cases in which criniviruses have been the underlying problem behind major epidemics even though they were not originally recognized as such. The examples of SPVD, strawberry decline, and BYVD illustrate how criniviruses can be asymptomatic in single infections and yet cause serious diseases in the presence of virus complexes with major impacts on plant health and yield. Furthermore, even criniviruses normally regarded as symptomatic can be asymptomatic in some hosts. Most members of the genus also require a minimum of 3 weeks for symptoms to become apparent. During this time infected plants can be moved to new areas or even new countries without evidence of infection. This fact has major implications at many levels; especially for viruses infecting clonally propagated crops (BPYV, BYVaV, PYVV, SPaV, and SPCSV) or crops associated with grafted transplants (CYSDV and CCYV). In today’s global trading environment there is constant germplasm exchange among individuals and organizations. The previous examples of crinivirus-driven epidemics should become lessons for the future and provide the impetus to improve plant certification schemes. This will facilitate increasing international trade in plant and plant products while decreasing the unintentional movement of plant pathogens. Given that some of the aforementioned viruses remain confined in specific geographic areas (i.e., BYVaV in the United States, PYVV in northwestern South America) it is still feasible to minimize their future impact by eliminating movement of infected material into areas where these viruses are not present. It is also important to establish vector exclusion strategies at the nursery or propagation field level. It has been common practice in certification schemes that plants are only visually inspected at the certified plant (G4) level. Using strawberry or blackberry as an example, neither BPYV, BYVaV nor SPaV cause symptoms in single infections in modern berry cultivars. However, when singly infected plants are planted in the field they often become infected with additional viruses and the resulting mixed infections can lead to serious epidemics. Exclusion and testing at the G4 level or prior to distribution can enhance longevity and profitability of the crops within regions and prevent or reduce accidental introduction of viruses into new production areas.

Given the relatively recent identification of criniviruses as economically important disease agents, work has primarily focused on characterization, epidemiology, and in certain cases chemical control of vectors. Still, the ultimate control strategy for any pathogen is strong, stable genetic resistance. Resistance using modern methods such as RNA interference is probably the most straightforward and durable approach to prevent infection by viruses, but public resistance to genetically modified plants especially in crops that are labeled as “healthy food” or “superfoods” such as fruits and vegetables, the primary hosts for criniviruses, has minimized the application of this technology. For the majority of the criniviruses little or no work has been directed toward identification of resistance using traditional screening of germplasm resources and/or breeding to incorporate such sources into commercially acceptable cultivars. In the few cases where resistance has been identified it is almost always found in wild accessions, which requires many generations of backcrossing before the relevant genes are incorporated into marketable varieties. That is not to say progress is not being made. Sources of resistance to LIYV were identified in both lettuce and melon (McCreight, 1987, 2000), although the demise of LIYV as an agricultural threat due to shifting vector population dynamics largely rendered advancement of the material a moot point. Other efforts however offer real potential for effective crinivirus management. A source of resistance to ToCV was recently identified in tomato (Garcia-Cano et al., 2010), and two independent and complementary sources of resistance to CYSDV have been found in melon (Lopez-Sesé and Gomez-Guillamon, 2000; McCreight and Wintermantel, 2011). Sequencing of the genomes of many crops affected by criniviruses, identification of resistance sources, and the use of marker-assisted selection will speed up the incorporation of these and likely other resistance traits into commercially relevant cultivars.

Criniviruses are transmitted in a semi-persistent manner and chemical control of vectors has not always been effective for virus disease management. In addition, the development of resistance to insecticides in insect populations and the effect of insecticides on whitefly predators may have a negative impact on vector and virus control, particularly in systems using broad integrated pest management approaches. Consequently, it may be appropriate to consider a more generic approach, such as identification of resistance against whitefly vectors. There have been several cases in which insect resistance has been identified in plants (Mutschler and Wintermantel, 2006). In many cases this has been more effective and long-lived than virus resistance, possibly due to the ability of the viruses to drift toward resistance-breaking populations. In addition, vector resistance may be effective in controlling several viruses that are transmitted by a common vector. As an extreme example, aphid resistance to *Amphorphora agathonica* (Hottes) had been effective for over 50 years in controlling three aphid-borne viruses in raspberry in the North America, before new biotypes of the vector developed that overcame the resistance (Hall et al., 2009). Forms of resistance against insects can function in a number of ways, including acting as feeding deterrents, physical barriers, or oviposition inhibitors. Some plant secondary metabolites dissuade insects from settling on plants, preventing the steady feeding that can lead to toxicity or virus transmission. Others may prevent oviposition, reducing vector populations (Mutschler and Wintermantel, 2006). Studies are just beginning to address the potential of resistance to insect feeding on control of whitefly-transmitted viruses (Mutschler and Wintermantel, 2006; Rodriguez-Lopez et al., 2011, 2012). Appropriate and effective utilization of such approaches will require specific research to confirm that methods effective in controlling one pest do not exacerbate problems with another. Integrating vector control with other means of pest and disease management; however, offers the potential to strengthen durability and effectiveness of control for not only criniviruses, but a number of insect-borne pathogens.

There have been numerous significant breakthroughs in understanding criniviruses, the diseases they cause, and their epidemiology. However, a great deal more work is needed on virus control, including an emphasis on certification to minimize virus movement, identification of resistance sources against both vectors and viruses, and introgression of resistance genes into commercially acceptable germplasm. These should be priority areas for long-term reliability of crinivirus management. Such efforts will complement or reduce the need for extensive pesticide-based programs, and will minimize the impact and spread of criniviruses in world agriculture.

## Conflict of Interest Statement

The authors declare that the research was conducted in the absence of any commercial or financial relationships that could be construed as a potential conflict of interest.
